# Whole-Body Cryotherapy Affects Blood Vitamin D Levels in People with Multiple Sclerosis

**DOI:** 10.3390/jcm14093086

**Published:** 2025-04-29

**Authors:** Bartłomiej Ptaszek, Szymon Podsiadło, Olga Czerwińska-Ledwig, Aneta Teległów

**Affiliations:** 1 Institute of Applied Sciences, University of Physical Culture in Krakow, 31-571 Krakow, Poland; 2 Institute of Clinical Rehabilitation, University of Physical Culture in Krakow, 31-571 Krakow, Poland; szymon.podsiadlo@awf.krakow.pl; 3 Institute of Basic Sciences, University of Physical Culture in Krakow, 31-571 Krakow, Poland; olga.czerwinska@awf.krakow.pl (O.C.-L.); aneta.teleglow@awf.krakow.pl (A.T.)

**Keywords:** whole-body cryotherapy, MS, vitamin D

## Abstract

**Background:** The aim of this study was to investigate and evaluate the effect of a series of 20 whole-body cryotherapy (WBC) treatments on the level of vitamin D in the blood of women with multiple sclerosis and healthy women. **Methods:** This study involved three participant groups. The experimental group included 15 women, aged 34 to 55 years and diagnosed with multiple sclerosis (MS), who received whole-body cryotherapy. The first control group comprised 20 women with MS who did not undergo cryotherapy. The second control group consisted of 15 women, aged 30 to 49 years, who did not have neurological or chronic conditions and who also participated in whole-body cryotherapy. Venous blood samples were taken from all participants at two points: on the first day of cryotherapy and after completing 20 sessions. These samples were analyzed to evaluate their key parameters and assess the differences between the groups. The electrochemiluminescence (ELISA) technique was employed using the 25(OH)D (total) competitive assay, which is designed to measure vitamin D concentration [ng/mL] in the human body. **Results:** In women with MS, a significant increase in vitamin D levels was observed after the use of WBC (CRYO-MS), while in healthy women the increase was statistically insignificant after WBC (CONTROL-CRYO). **Conclusions:** After 20 whole-body cryotherapy sessions, an increase in vitamin D levels was observed in women with multiple sclerosis. A trend towards an increase in vitamin D levels was observed in healthy women after WBC.

## 1. Introduction

Currently, it is believed that multiple sclerosis (MS) is related to a combination of hereditary predispositions and currently undefined environmental factors [1]. The environmental factors determining MS have not been clearly defined. Studies suggest that MS may be the result of a viral infection, but the potential virus has not been identified. Environmental factors that may be indirectly related to the occurrence of the disease are also being studied. The argument that environmental factors significantly influence the risk of morbidity is supported by the varying incidence rates of multiple sclerosis (MS) in different geographic regions. Studies examining the relationship between MS and environmental influences have considered a range of factors, including geoclimatic and seasonal conditions, and economic, social, racial, and ethnic variables [2,3].

Hematological changes in patients with MS are not specific or characteristic, making it challenging to identify a uniform underlying mechanism. In fact, such a mechanism may not exist. It has been suggested that these changes could be associated with various factors, including complex humoral and cellular immune responses, biochemical imbalances, alterations in vitamin B12 binding and transport, the effects of pharmacological treatments, and different phases of the disease. Analyzing previous reports on the effect of whole-body cryotherapy (WBC) in MS patients, the authors found that WBC can increase patients’ TAS (total antioxidant status) and may play an important role in the process of activating antioxidant systems in MS patients, but no significant changes in antioxidant enzymes (catalase, glutathione peroxidase) were detected, except for in superoxide dismutase, when the number of WBC sessions was increased to 30, suggesting that different cooling interventions may influence the changes seen in antioxidant capacity [4,5,6,7,8,9]. Moreover, WBC does not negatively affect RBC deformability and aggregation, making it safe for MS patients [10]. When assessing the psychological parameters and functional performance of patients, it is worth noting that WBC can lead to reduced fatigue and improved functional status, potentially improving the general well-being, mood symptoms, depression, and quality of life of the subjects [11,12,13,14]. These changes may be due to adaptive changes in muscle bioelectric activity [15].

Among the external factors most often associated with the pathogenesis of MS are bacterial and fungal infections and vitamin D deficiency. The distribution of MS’s incidence in the world is correlated with exposure to sunlight and the amount of vitamin D synthesized by the body. It has been found that low concentrations of vitamin D in humans (hypovitaminosis D) are associated with the more frequent occurrence of MS. Studies of vitamin D concentration in the serum of MS patients have shown that its deficiency is present in the majority of patients and also in the earliest stages of the disease [16]. A similar relationship has been observed in several other autoimmune diseases [17]. Modern molecular techniques have made it possible to determine that the action of vitamin D may mainly occur through the functioning of its receptor, which seems to be not only a mediator of biological action but also a mediator of the regulation of the metabolism of this vitamin [18]. It is known that some cells of the immune system express vitamin D receptors and that the vitamin and its analogs have a profound modulating effect on the immune system and probably MS. It is worth noting that despite the increasing number of studies on the effects of WBC on the human body, the potential impact of cryotherapy on the body’s vitamin D status remains undetermined. This is particularly notable given the well-established anti-inflammatory, immunomodulatory, antioxidant, and antifibrotic properties of vitamin D [19].

In our study, we aimed to investigate the effect of repeated exposure to whole-body cryotherapy at −120 °C in healthy and MS individuals on their 25(OH)D levels. The response to cold was assessed before and after a series of 20 treatments. Given that the incidence rate of MS in women relative to men is approximately 2:1, we recruited women for this study.

## 2. Materials and Methods

### 2.1. Participant Characteristics

This (controlled and prospective) study was conducted in accordance with the Helsinki Declaration of the World Medical Society, with consent from the Bioethics Committee at the Regional Medical Chamber in Krakow (87/KBL/OIL/2018; 8 May 2018) and registered in the Australian New Zealand Clinical Trials Registry (ACTRN12620001142921; 2 November 2020). Participants passed a medical assessment (performed by a neurologist and rehabilitation physician) before participating in this project.

To take part in this study, volunteers had to have been diagnosed with MS (McDoald’s review criteria), obtain an Extended Disability Status Scale (EDSS) score from 0 to 6.5, be a woman, be aged 30–55 years, and sign a consent form.

Volunteers could not have contraindications to WBC (i.e., pregnancy, severe hypertension (BP > 180/100), acute or recent myocardial infarction, unstable angina pectoris, arhythmia, symptomatic cardiovascular disease, cardiac pacemakers, peripheral arterial occlusive disease, venous thrombosis, an acute or recent cerebrovascular accident, uncontrolled seizures, Raynaud’s Syndrome, fever, tumor disease, symptomatic lung disorders, bleeding disorders, severe anemia, infection, cold allergy, and acute kidney and urinary tract diseases); change diets during the project or within the 3 months before the project; or take part in other forms of treatment or physical activity during the project or in the 3 months before it.

A total of 50 women were enrolled in this study and divided into three groups:

- CRYO-MS (study group): Fifteen women aged 34 to 55 years diagnosed with multiple sclerosis (MS) who underwent 20 WBC treatments.
- CONTROL-MS (first control group): Twenty women aged 32 to 48 years diagnosed with multiple sclerosis who did not receive WBC treatments. This group was selected through nonprobability sampling and included patients who had no contraindications to WBC but were unable to participate due to various constraints (such as work or family obligations).
- CONTROL-CRYO (second control group): Fifteen healthy women aged 30 to 49 years without any chronic diseases, including neurological conditions, who also underwent 20 WBC treatments.

### 2.2. Analysis of Vitamin D Levels

For the analysis of blood indices, venous blood samples were collected at two time points: at baseline, on the day the whole-body cryotherapy (WBC) treatments commenced (study 1), and after 20 WBC treatments (study 2). Participants in the control group who did not undergo cryotherapy had their blood drawn only once. During the study, blood pressure was monitored in patients receiving cryotherapy both before and after each WBC session.

Blood samples were collected in the morning while the participants were fasting, using either the antecubital, cephalic, or median vein. The samples were placed in test tubes containing a clotting activator, specifically SiO_2_ (6 mL), for serum analysis. The blood parameters were analyzed at an accredited laboratory. The level of vitamin D was determined using the DRG 25 (OH) Vitamin D (total) ELISA Kit, with readings taken at 450 nm (DRG Instruments GmbH, Marburg, Germany). The electrochemiluminescence method was employed and utilized a competitive test for 25 (OH) D (total) to measure the vitamin D levels in the blood, which were expressed in ng/mL.

### 2.3. Description of the Intervention

WBC sessions were conducted at the Malopolska Center of Cryotherapy and Rehabilitation in Krakow. The treatment parameters included an atrium temperature of −60 °C and a chamber temperature of −120 °C, with liquid nitrogen utilized as the cooling refrigerant. The duration of a single WBC session varied based on the treatment stage: 1.5 min for the first session, 2 min for the second session, and 3 min for sessions three through twenty. Patients participated in treatments once daily; these were scheduled between 15:00 and 17:00, with a total of five sessions per week, culminating in 20 treatments. Each cryotherapy session was followed by a 15 min warm-up on a cyclo-ergometer, during which no resistance was applied.

### 2.4. Statistical Analysis

In order to determine the sample size, the formula for the minimum sample size was used, in which the confidence interval was 95%, the fraction size of 0.5, and a maximum error of 5% was assumed. Data were presented as mean values with standard deviations. The normality of their distributions was assessed using the Shapiro–Wilk test. Differences between the experimental group and the control groups were analyzed with a one-way analysis of variance (ANOVA) or, when the assumptions for an ANOVA were not met, the Kruskal–Wallis test. For our post hoc analysis, either Tukey’s test for unequal sample sizes or the multiple comparison test of mean ranks for Dunn’s trials was utilized. Dependent variables were assessed with a paired Student’s *t*-test; if its assumptions were not satisfied, the Wilcoxon test was applied instead. Independent variables underwent evaluation using the Mann–Whitney U test. A significance threshold of α = 0.05 was established for all tests, which were performed to assess two-tailed hypotheses. Statistical computations were carried out using the Statistica 13 software package (StatSoft, Dell Inc., Round Rock, TX, USA).

## 3. Results

A significant increase in vitamin D levels was noted in women with MS after the use of WBC (CRYO-MS) (on average by 11.85 ng/mL) and a statistically insignificant increase was noted in healthy women after WBC (CONTROL-CRYO) (on average by 2.19 ng/mL). When analyzing the patients’ baseline levels of vitamin D, higher levels were noticeable in women with MS from the study group and the control group compared to healthy women. However, these differences were not statistically significant (*p* = 0.339 and *p* = 0.052, respectively) (Table 1, Table 2 and Table 3).

## 4. Discussion

Vitamin D deficiency has been associated with the onset, susceptibility, relapse rate, disability, and disease progression of MS [20,21,22,23,24]. Epidemiological data support a correlative association between vitamin D deficiency and/or sunlight exposure in early life and an increased risk of developing MS in adulthood [25,26]. Vitamin D has also been found to be a protective factor against MS; low serum vitamin D levels correlate with an increased risk of developing MS [27,28]. According to the current literature, vitamin D is involved in the regulation of the course of MS. This suggests lower serum 25(OH)D3 levels during relapse in MS patients [29,30,31,32,33]. Furthermore, higher serum 25(OH)D3 levels have been found to have protective effects on the risk of MS, such as reducing CNS damage and relapse rates [34,35]. The exact mechanism of action of vitamin D in MS remains unclear, but it is likely due to its immunomodulatory effects on CNS inflammation [36]. In our study, we observed a significant increase in vitamin D levels in women with MS after WBC, and a statistically insignificant increase in healthy women after WBC. When analyzing their baseline levels, higher levels are noticeable in women with MS from the study group and the control group compared to healthy women. It seems that WBC may have a modulating effect on the level of vitamin D in healthy people and in MS.

Low serum 25(OH)D3 levels (around 20 ng/mL) are usually observed in MS patients even at the beginning of the disease [35,37,38,39]. However, normal serum levels (above 30 ng/mL) can also be observed in patients, as the disease is multifactorial. A vitamin D deficiency in MS patients can be partly explained by the abnormalities in vitamin metabolism occurring in patients [40]. In the later course of the disease, serum vitamin D levels show a clear tendency to become even more reduced. Two related factors may contribute to this reduction: first, the Uhthoff phenomenon, in which patients tend to avoid direct sunlight because the heat worsens their symptoms; second, disability leads to less outdoor activity and consequently less exposure to UVB [41]. Immunological mechanisms play a major role in the pathogenesis of MS. Smolders et al. (2009) observed that higher 25(OH)D levels are associated with the increased functionality of regulatory T cells and a higher Th2 to Th1 ratio, which results in the resolution of inflammation and the inhibition of the autoimmune response [42].

In patients already diagnosed with the disease, vitamin D probably has a protective effect and stabilizes the patient’s condition. Smolders et al. (2008) showed that in patients with relapsing–remitting MS, who had been ill for no longer than 5 years, higher concentrations of 25(OH)D were correlated with a reduced risk of disease relapse [43]. Similar results were obtained by Pierrot-Deseilligny et al. (2012), who showed that every 10 nmol/L increase in blood 25(OH)D concentration was associated with a 13.7% reduction in the risk of disease relapse [44]. In the study conducted by Simpson et al. (2010) in a group of patients with relapsing–remitting MS, an inverse correlation was also observed between the blood 25(OH)D concentration and the risk of disease relapse—every 10 nmol/L increase in blood 25(OH)D concentration was associated with a 12% reduction in the risk of disease relapse [34]. An inverse correlation between the concentration of 25-hydroxyvitamin D in the blood and the risk of MS relapse was also observed by Soilu-Hanninen et al. (2005)—patients in a period of disease relapse had lower concentrations of 25(OH)D in their blood than patients in a period of remission [32]. In the studies conducted in this paper, a statistically significant increase in the level of vitamin D was noted in women with MS after the use of WBC (on average by 11.85 ng/mL) and a statistically insignificant increase was noted in healthy women after WBC (on average by 2.19 ng/mL). However, when analyzing their baseline levels, higher levels of vitamin D are noticeable in women with MS from the study group and the control group compared to healthy women. Nevertheless, these differences were not statistically significant (*p* = 0.339 and *p* = 0.052, respectively).

Recent studies have shown that vitamin D plays a significant role in preventing not only bone diseases but also cardiovascular diseases, cancers, muscle atrophy, and some mental disorders [45,46]. Pilch et al. (2017) [47], in their study on the effect of a six-week Nordic walking training program on healthy women over 55 years of age, observed a decrease in 25(OH)D concentration. The authors suggest that this change could be the result of the reduced biosynthesis of vitamin D by the skin due to participants’ lower exposure to UV radiation or due to the involvement of vitamin D in muscle metabolism [45,47,48]. Vitamin D deficiency is associated with increased inflammation and it has been shown that vitamin D3 supplementation can inhibit the release of CRP, TNF-α, and IL-6 [49]. Studies have also shown higher vitamin D levels at low IL-6 concentrations.

Correale et al. (2009) [33] observed an inverse correlation between the concentration of both vitamin D metabolites in the blood, 25(OH)D and 1,25(OH)2D, and the risk of relapse. In patients with relapsing–remitting MS, the concentrations of 25(OH)D and 1,25(OH)2D in their blood serum were higher during remission than during relapses, which suggests a potential beneficial effect of vitamin D on the course of the disease [33]. Smolders, in contrast to Correale, confirmed the effect of high 25(OH)D concentrations on the reduced risk of MS relapse, but did not observe such a relationship for 1,25(OH)2D [43]. No significant correlation was found between 25(OH)D and the IgG index and MRI changes [32,50]. In Tasmanian patients with a longer history of disease, reduced 25(OH)D levels were strongly correlated with increased EDSS scores [51].

The aim of Śliwicka et al.’s (2020) [52] study was to investigate the effects of single and repeated exposures to WBC in volunteers with different levels of physical fitness on 25-hydroxyvitamin D (25(OH)D). The study involved 22 healthy male volunteers who underwent 10 consecutive sessions in a cryogenic chamber once daily. After 10 cryotherapy sessions, 25(OH)D levels increased in the high-fitness group and decreased in the low-fitness group. Unfortunately, the changes were small and transient. The authors found that the body’s response to a series of 10 cryotherapy sessions is modified by a person’s level of physical fitness [52].

The observed changes in 25(OH)D levels appear to be linked to the role of vitamin D in modulating the inflammatory response. In support of this, Śliwicka et al. reported an inverse relationship between changes in 25(OH)D concentrations and hsCRP levels in their study. This aligns with existing research indicating that vitamin D possesses natural antioxidant and anti-inflammatory properties [52]. The authors also observed positive correlations between changes in 25(OH)D levels and myostatin (Mst) concentrations 30 min after the tenth WBC session, compared to baseline values. Additionally, negative correlations between changes in 25(OH)D and irisin levels within the group suggest a potential role for vitamin D in skeletal muscle metabolism [52]. Supporting this, Slivka et al. (2011) reported that cold-induced exercise increases the expression of the peroxisome proliferator-activated receptor gamma coactivator 1-alpha (PGC-1α) gene [53]. Irisin, which is secreted in response to PGC-1α [54], may function as a coactivator of the vitamin D receptor within mitochondria [55]. Moreover, Garcia et al. (2011) demonstrated that vitamin D inhibits Mst expression while promoting the expression of follistatin and insulin-like growth factor II, further indicating its involvement in muscle regulation [56].

In conclusion, considering the existing literature and our own research findings, it can be concluded that WBC is an effective approach for combating or slowing the progression of various diseases and mitigating their adverse effects, playing a crucial role in maintaining optimal physical fitness. It should be noted that the observed changes in serum 25(OH)D were small; therefore, MS patients may use WBC as an additional therapy. However, to fully understand the body’s response to WBC, additional studies with larger and more diverse study groups are necessary. It would also be beneficial to conduct a double evaluation of the non-WBC group and assess the long-term effects of the treatment.

## 5. Conclusions

After 20 whole-body cryotherapy sessions, an increase in vitamin D levels was observed in women with multiple sclerosis. A trend towards an increase in vitamin D levels was observed in healthy women after WBC as well. It seems that WBC may have a modulating effect on the level of vitamin D in healthy people and those with MS.

## Figures and Tables

**Table 1:** Vitamin D levels before the intervention sessions.

Parameter	CRYO-MS (*n* = 15)	CONTROL-MS (*n* = 20)	CONTROL-CRYO (*n* = 15)	*p* (ANOVA)
Vitamin D [ng/mL]	34.97 (28.05–46.92)	37.48 (29.06–78.09)	25.56 (22.56–37.08)	0.046 *
	*p* (CRYO-MS/ CONTROL-MS) 0.592	*p* (CONTROL-MS/ CONTROL-CRYO) 0.339	*p* (CRYO-MS/ CONTROL-CRYO) 0.052	

CRYO-MS: the group of women with multiple sclerosis (MS) who engaged in a series of whole-body cryotherapy (WBC) sessions; CONTROL-MS: the first control group, consisting of women with MS who did not undergo any cryotherapy treatments; CONTROL-CRYO: the second control group, composed of healthy women who participated in WBC sessions; ANOVA: analysis of variance; * indicates statistical significance (*p* < 0.05).

**Table 2:** Vitamin D levels after the intervention sessions.

Parameter	CRYO-MS (*n* = 15)	CONTROL-MS (*n* = 20)	CONTROL-CRYO (*n* = 15)	*p* (ANOVA)
Vitamin D [ng/mL]	46.82 (27.63–64.51)	37.48 (29.06–78.09)	27.75 (20.19–35.86)	0.021 *
	*p* (CRYO-MS/ CONTROL-MS) 0.987	*p* (CONTROL-MS/ CONTROL-CRYO) 0.056	*p* (CRYO-MS/ CONTROL-CRYO) 0.039 *	

CRYO-MS: the group of women with multiple sclerosis (MS) who engaged in a series of whole-body cryotherapy (WBC) sessions; CONTROL-MS: the first control group, consisting of women with MS who did not undergo any cryotherapy treatments; CONTROL-CRYO: the second control group, composed of healthy women who participated in WBC sessions; ANOVA: analysis of variance; * indicates statistical significance (*p* < 0.05).

**Table 3:** Changes in vitamin D levels after the intervention sessions.

Parameter	Before	After	*p* (*t*-Student/Wilcoxon)
CRYO-MS (*n* = 15)			
Vitamin D [ng/mL]	34.97 (28.05–46.92)	46.82 (27.63–64.51)	0.013 *
CONTROL-CRYO (*n* = 15)			
Vitamin D [ng/mL]	25.56 (22.56–37.08)	27.75 (20.19–35.86)	0.759

CRYO-MS: the group of women with multiple sclerosis (MS) who engaged in a series of whole-body cryotherapy (WBC) sessions; CONTROL-MS: the first control group, consisting of women with MS who did not undergo any cryotherapy treatments; CONTROL-CRYO: the second control group, composed of healthy women who participated in WBC sessions; ANOVA: analysis of variance; * indicates statistical significance (*p* < 0.05).

## Data Availability

All data generated or analyzed during this study are included in the published article.
